# Implementation Science Application to EEG Neurofeedback Research: A Call to Action

**DOI:** 10.15540/nr.11.2.211

**Published:** 2024-06-27

**Authors:** Whitney K. Norris, M. Kathryn Allison, Sebern Fisher, Geoffrey M. Curran

**Affiliations:** 1 University of Arkansas for Medical Sciences, Little Rock, Arkansas, USA; 2 Private Practice, Northampton, Massachusetts, USA

**Keywords:** neurofeedback, implementation science, systematic review

## Abstract

This article is a call to action for implementation research in the field of electroencephalogram (EEG) neurofeedback. While the effectiveness of neurofeedback in improving clinical outcomes has been well established and is continuing to expand into a variety of symptom presentations and mechanisms of action, there is lack of research bridging the gap between the research setting and neurofeedback’s implementation in mental health clinics. Our review of the published research to date revealed no articles incorporating the burgeoning utility of implementation science into neurofeedback research to bridge the gap and provide practical information about how to use neurofeedback in real-world settings. Research is urgently needed to explore the feasibility and process of implementing neurofeedback in the clinical setting, without which the applicability and usefulness of outcome studies are called into question.

## Introduction

Electroencephalogram (EEG) neurofeedback first began to gain popularity in the 1950s and 1960s as an intervention to treat epilepsy and anxiety disorders (Hardt & Kamiya, 1978; Sterman & Friar, 1972). Since this time, the utilization of neurofeedback has branched into a wide variety of symptoms and treatment goals within and outside of the mental health field. A 2016 comprehensive review of the literature by the International Society of Neuroregulation and Research (ISNR, formerly the International Society for Neurofeedback and Research) found over 700 published articles related to the use of neurofeedback to treat a wide range of presenting issues and disorders from attention-deficit/hyperactivity disorder (ADHD) to performance enhancement (Hammond & Novian, 2017). Since the publication of that review, a review of neuromodulation research was published in 2019, which reviewed the ISNR bibliography and highlighted a wide variety of studies across different types of neurofeedback (Perl & Perl, 2019). This review found 314 total studies exploring the effects of neurofeedback. To date, neurofeedback has been found to have sustained effects in the treatment of ADHD (Arnold et al., 2021; Bluschke et al., 2020; Dobrakowski & Łebecka, 2020; Lubar & Shouse, 1976; Purper-Ouakil et al., 2019; van Doren et al., 2019), reduction of symptoms and reduced relapse rates in substance use disorder (Dalkner et al., 2017; Dehghani-Arani et al., 2010, 2013; Goldberg et al., 1976; Horrell et al., 2010; Ko & Park, 2018; Lackner et al., 2016; Lamontagne et al., 1975; Passini et al., 1977; Peniston & Kulkosky, 1989; Saxby & Peniston, 1995; Scott et al., 2005), and a significant decrease in posttraumatic stress disorder (PTSD) symptoms (Fisher et al., 2016; Gapen et al., 2016; Kluetsch et al., 2014; Leem et al., 2021; Nicholson et al., 2020; Noohi et al., 2017; Peniston & Kulkosky, 1991; Rogel et al., 2020), including participants who had not responded well to previous PTSD treatment (Askovic et al., 2020; van der Kolk et al., 2016). After this success in the utilization of neurofeedback to treat PTSD, more recent research has also explored the specific mechanisms of neurofeedback that are having an impact, including changes to the brain’s default mode network, a commonly implicated network in trauma psychopathology (Bluhm et al., 2009; Kluetsch et al., 2014; Lanius et al., 2015; Nicholson et al., 2020). This forward movement in the field of PTSD was also substantiated in 2023 by FDA clearance being granted to a neurofeedback for the treatment of PTSD (GrayMatters Health, 2023).

While a variety of individual studies have illustrated the efficacy of specific types of neurofeedback and neurofeedback protocols in treating the above mentioned mental health conditions, the neurofeedback field has struggled to establish as robust of an evidence-base as other interventions in the mental health field due in part to the wide variety of neurofeedback approaches, inconsistent research design to allow for more across study comparisons and meta-analyses (i.e., lack of controls and debate over the use of sham conditions), and insufficient funding to support more large-scale randomized controlled trials (Fisher et al., 2016; Kuznetsova et al., 2022; Marzbani et al., 2016; Micoulaud-Franchi et al., 2021; Perl & Perl, 2019; Riesco-Matías et al., 2021; Trocki, 2006; van der Kolk et al., 2016). Despite claims regarding neurofeedback’s effectiveness comparability with “gold standard” mental health treatments (Nicholson et al., 2020), some within the mental health field question its credibility in part due to its controversial history, which includes significant disagreements and contradictions from key stakeholders in the field (Kuznetsova et al., 2022; Robbins, 2008). Neurofeedback clinicians have questioned why neurofeedback is not more popular in the mental health field based on the results they see in their practices (Robbins, 2008). While some have speculated as to why neurofeedback is not more commonly implemented in mental health settings (i.e., expense, lack of insurance coverage, theoretical differences among practitioners; Marzbani et al., 2016; Robbins, 2008), the field lacks scientific evidence to establish these and other potential factors as implementation barriers.

As any research-aware clinician knows, discovering what interventions work in the laboratory setting is not enough to successfully bring them to the clinical settings (Bauer & Kirchner, 2020). It is not uncommon in the mental health field to find interventions that have been shown to be effective in the literature that are not commonly or correctly utilized in actual clinical practice (Kettlewell, 2004). In a commentary on the topic of dissemination and implementation of evidence-based treatments in the field of mental health, Kettlewell pointed to a gap between science and clinical practice in psychology as a significant problem for the field and that our ability to close this gap “will determine our ability to remain a highly regarded helping profession” (Kettlewell, 2004, p. 190). He went on to state, “We have treatments that work, and most practitioners do not use them” (Kettlewell, 2004, p. 190). As much as 85% of medical research dollars do not impact the public due to what is referred to as “research waste” (Chalmers & Glasziou, 2009). The NIMH has the strategic goal to “speed up the development, adoption, and implementation of effective, evidence-based mental health services to improve the reach and outcomes of these services in diverse communities and populations” (National Institute of Mental Health, 2022). For research to have the desired impact, it is imperative that we identify feasible implementation strategies that minimize the barriers to implementing evidence-based interventions in real-world settings. Therefore, research using implementation science approaches is needed to optimize implementation of evidence-based interventions like neurofeedback.

Implementation science is the scientific study of how evidence-based clinical interventions or research findings are best adopted and integrated into routine practice (Eccles & Mittman, 2006). Recognizing that many evidence-based interventions either never make it into routine care or take many years to do so (Balas & Boren, 2000; Morris et al., 2011), implementation science offers methods to identify factors that influence uptake and sustained use of evidence-based mental health interventions and strategies that support implementation. Clinical implementation is an important step in the translational science spectrum, as it bridges the gap between clinical research findings and integration into routine care for the general public. Because there is strong evidence for the clinical effectiveness of EEG neurofeedback (Markiewcz, 2017), implementation research in mental health care settings is the logical next step.

## Approach and Findings

To explore the extent of research on the implementation of neurofeedback in mental health settings, we conducted a systematic literature review. We focused on peer-reviewed articles published since 1995 that explored implementation factors, strategies, and/or outcomes. After several informal searches provided no relevant articles, we partnered with a Health Sciences Informationist at the University of Arkansas for Medical Sciences (UAMS) to conduct a review of four databases (PubMed, PsycInfo, CINAHL, SocINDEX) using the following search terms: (“neurofeedback” OR “EEG neurofeedback” OR “EEG biofeedback” OR “biofeedback”) AND (“Implementation Science research” OR “Implementation Science framework” OR “implementation science” OR “implementation research” OR “knowledge translation”). This search resulted in no articles exploring the use of an implementation science lens to explore the utilization of EEG neurofeedback in mental health treatment.

Despite many clinicians’ and researchers’ support of the use of neurofeedback, our search identified no implementation peer-reviewed research articles to date that specifically or systematically explored the barriers and facilitators of neurofeedback’s use in clinical mental health settings or strategies needed to promote uptake or sustain use in these settings. The closest relevant studies found in our informal search were two studies that broadly explored neurofeedback practitioners’ perspectives (Larson et al., 2010; Luctkar-Flude et al., 2019) and one that explored the experiences of neurofeedback clients (Aguilar-Prinsloo & Lyle, 2010). Luctkar-Flude et al.’s (2019) study specifically explored neurofeedback clients’ and providers’ experiences of the results of neurofeedback in a very specific client population—cancer survivors. While this was a well-designed study that provided useful insights, the author’s goal was to describe the personal experiences and outcomes of those involved in the neurofeedback treatment, not specifics regarding the barriers and facilitators to its implementation.

Larson and colleagues’ (2010) study came a step closer to the implementation research we are suggesting here. While the authors did not approach the study with an implementation science lens, part of their goal was to learn more about neurofeedback providers’ beliefs about aspects of the use of neurofeedback, such as advantages, disadvantages, and other components of using neurofeedback in a mental health setting. Larson et al.’s (2010) study resulted in three main findings: the effectiveness of neurofeedback in treating a variety of mental health issues, the need for extensive practitioner commitment due to the complexity of the intervention, and problems related to dissemination and funding of neurofeedback. Though practitioners’ beliefs about an intervention are one of many potential determinants of implementation, there are many other potential determinants to explore for a full understanding of implementation factors (Damschroder et al., 2009). Therefore, Larson et al.’s (2010) exploration is only the tip of the iceberg in understanding implementation factors for neurofeedback in mental health settings. Additional research is needed to explore all possible determinants of neurofeedback implementation in order to make this evidence-based intervention more accessible to the public.

## Future Research

**Figure 1.**
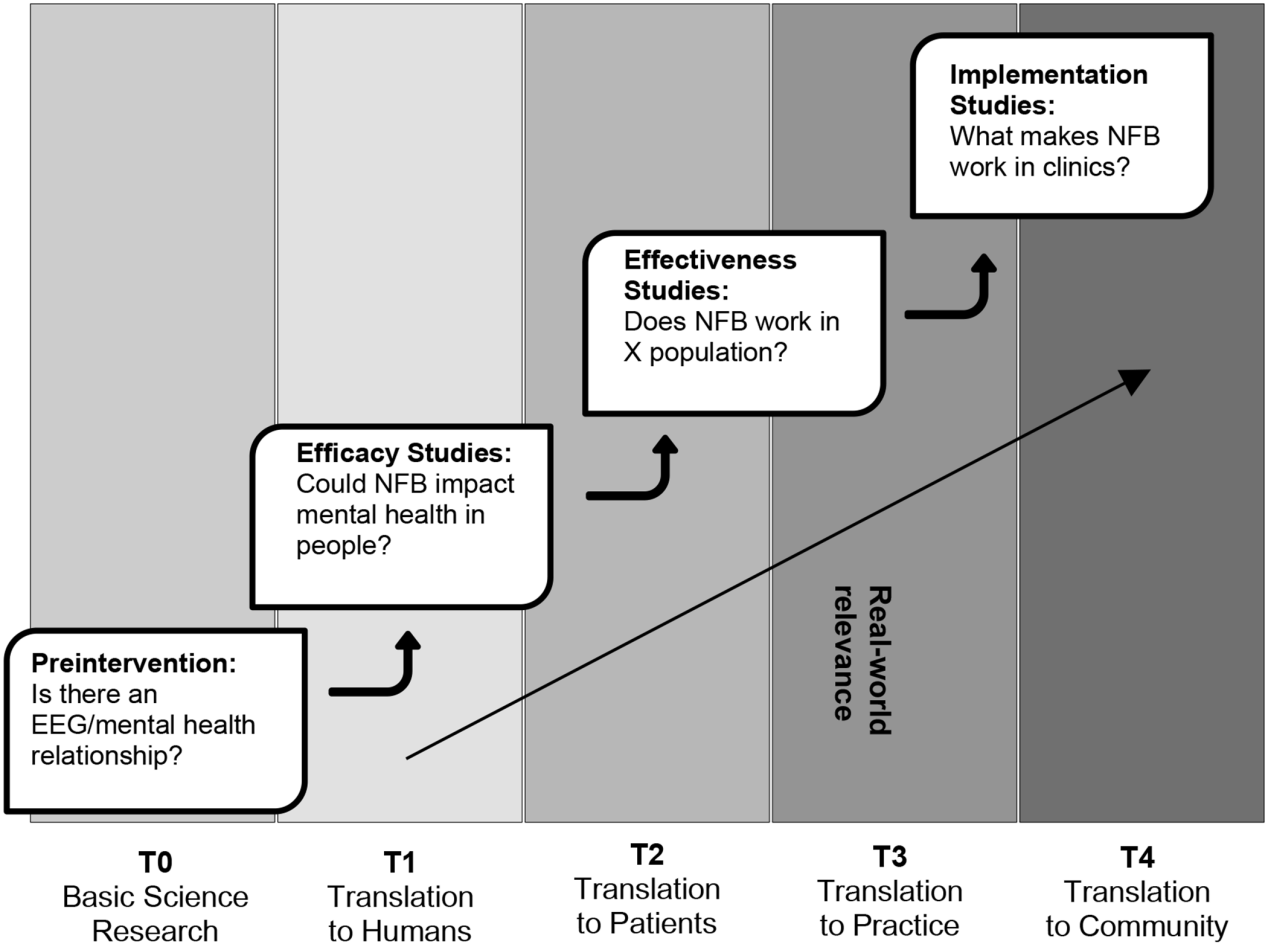
Translational Research Continuum.

With evidence for the effectiveness of neurofeedback in treating a variety of mental health conditions, the natural next step in the translational research continuum “from bench to bedside” (Drolet & Lorenzi, 2011) is to further study the implementation of neurofeedback to increase its delivery in routine mental health care. Figure 1 illustrates how the translational research continuum applies to research in the neurofeedback field. As mentioned above, the work to date from preintervention studies to studies of efficacy and effectiveness of neurofeedback have shown that neurofeedback can and does impact mental health within a variety of treatment settings and symptoms presentations. However, our larger point here is that this last phase of the research continuum involved in illustrating the real-world relevance of the intervention is lacking and represents the next step in the application of neurofeedback in mental health treatment.

Within the field of implementation science research, there are a wide variety of models, frameworks, and approaches that provide tangible insight into barriers and facilitators to intervention implementation across different settings and from the perspectives of different stakeholders throughout the process (Brownson et al., 2017). The EPIS model is one worthwhile example of a model that can be used to organize and provide specific direction for future neurofeedback implementation research (Aarons et al., 2011).

**Figure 2.**
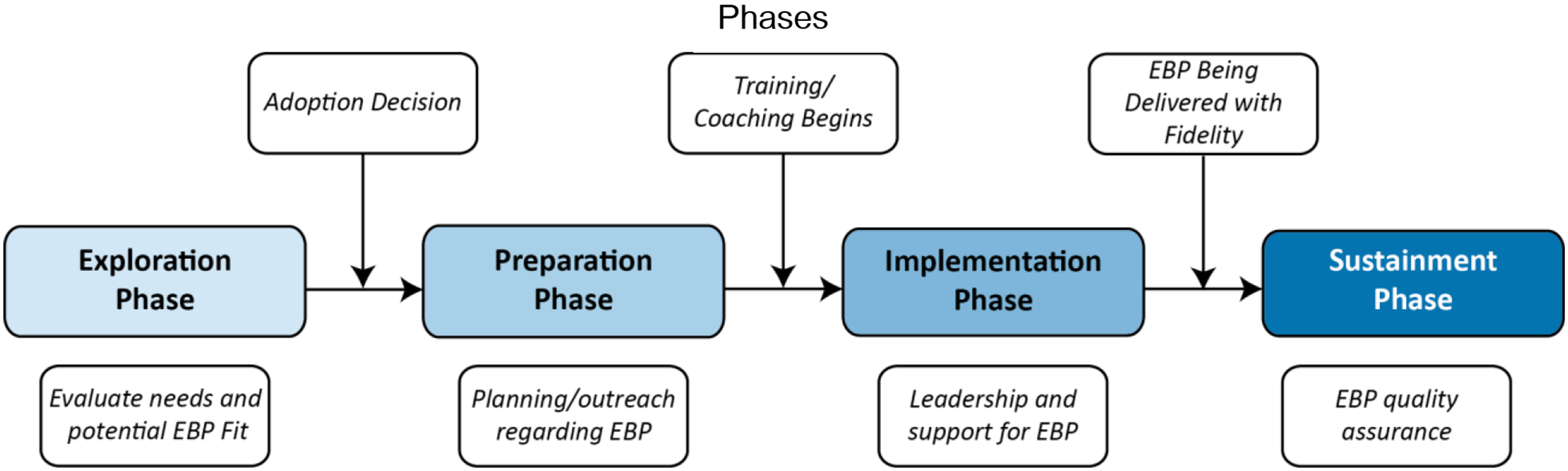
The Four Phases of the EPIS Model (EPIS Framework, 2024).

**Figure 3.**
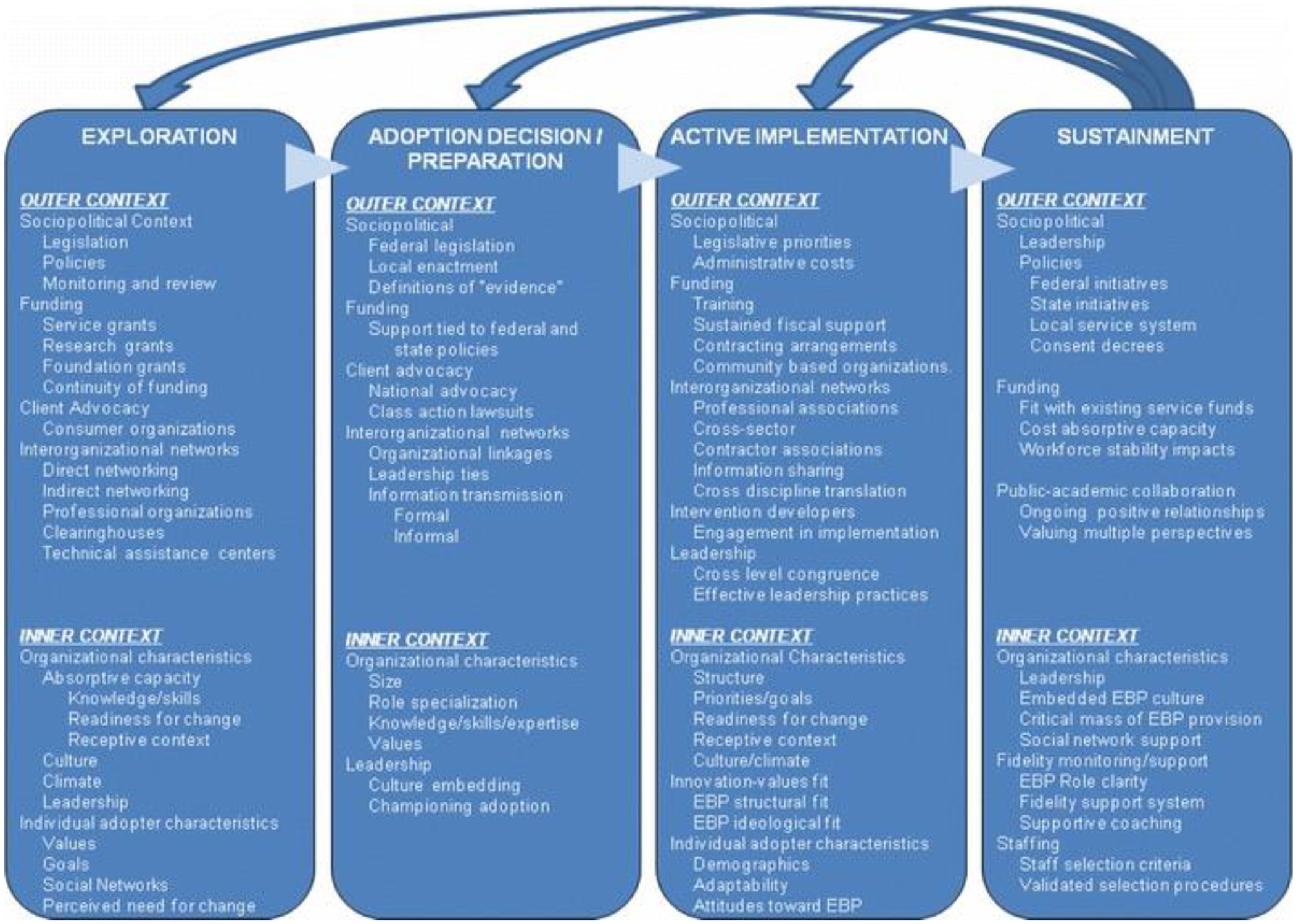
Aarons et al. (2011) Conceptual Model of Implementation Phases and Factors Affecting Implementation in Public Service Sectors.

The EPIS model describes four phases of the implementation process and specific elements within each of these phases that past research has shown to have an impact on evidence-based practice (EBP) implementation (see Figure 2). These phases are meant to evaluate current needs and evaluate EBP fit before the implementation is adopted (the Exploration phase), planning and outreach regarding the EBP (the Preparation phase), early active implementation of the EBP (the Implementation phase), and finally, sustained implementation and possible adaptation of the EBP over the long term (the Sustainment phase). Each phase includes inner and outer context factors, as well as factors that bridge these contexts. For example, outer context factors in the neurofeedback field could include professional organizations’ support for neurofeedback, research funding to support randomized-controlled trials of neurofeedback in mental health settings, or insurance panel advocacy for coverage of neurofeedback as part of routine mental health care (see Figure 3). Inner context factors could include variables such as organization and individual practitioner characteristics, staffing structures of specific mental health clinics, and the perceived need for change or the addition of an intervention like neurofeedback in the clinics’ clinical practice offerings.

Due to the scarcity of literature on neurofeedback implementation described above, future neurofeedback implementation research could benefit from focusing on any variety of the specific contextual factors within any of the four EPIS phases. One strategy to discern which of these factors or areas on which to focus would be to learn from experienced neurofeedback practitioners, consultants, and trainers to learn more about their anecdotal observations of the barriers and facilitators of implementation of neurofeedback. Two possible examples are described below:

- An experienced neurofeedback practitioner observes a trend among new neurofeedback providers who, initially, are excited to start using neurofeedback but give up within 6 months. This insight could lead to an exploration of inner and/or outer context factors with the Sustainment phase of the EPIS. The use of longitudinal surveys and/or interviews of newly trained neurofeedback practitioners could provide insight into what specific factors are leading to this failure of implementation, which could lead to tangible changes in the training or early mentoring of neurofeedback practitioners.
- Neurofeedback practitioners may observe a general lack of knowledge within the mental health field of the use of neurofeedback in mental health treatment. This could lead to research within the Exploration phase by surveying individuals, organization leaders, and other stakeholders about their knowledge of neurofeedback and/or the process involved in how they usually learn about new interventions that they may later adopt.

As in the early phases of any new research, the exploration must begin somewhere. The breadth and depth provided by an established field like implementation science allows for the possibility of building a strong foundation that can be built upon for many years to come. Much like the study of the brain, as the field of neurofeedback implementation research blossoms and grows, the answers we find will likely lead to even more questions, which will lead to a deeper and richer understanding that will benefit the field greatly.

## Conclusion

In describing the barriers to translating research into practice in critical care, Berenholtz and Pronovost said, “the most cost-effective opportunity to improve patient outcomes will likely come not from discovering new therapies but from discovering how to deliver therapies that are known to be effective” (Berenholtz & Pronovost, 2003, p. 321). We believe that neurofeedback is an ideal intervention to improve patient outcomes in this way. Anecdotally, we have heard that there may be concerns in the field about a lack of public and healthcare community awareness about neurofeedback, the costs associated with neurofeedback to both clinician and client, and other provider- and clinic-level barriers; however, without implementation research to systematically identify these issues, it is unknown which are true barriers and what implementation strategies are needed to overcome them. While the outcome studies are promising, without a clear understanding of how to bring this intervention to these populations, including the barriers and facilitators to doing so, the research will be of little use. It is time for the field of neurofeedback to convert its abundance of successful outcomes research and so many neurofeedback providers’ and clients’ countless “n of 1” experiences (S. Fisher, personal communication, August 10, 2022; Panisch & Hai, 2020) into tangible application through the use of established frameworks, like EPIS, provided by implementation science.
